# Diverse RNA Viruses Associated with Diatom, Eustigmatophyte, Dinoflagellate, and Rhodophyte Microalgae Cultures

**DOI:** 10.1128/jvi.00783-22

**Published:** 2022-10-03

**Authors:** Justine Charon, Tim Kahlke, Michaela E. Larsson, Raffaela Abbriano, Audrey Commault, Joel Burke, Peter Ralph, Edward C. Holmes

**Affiliations:** a Sydney Institute for Infectious Diseases, School of Life and Environmental Sciences and School of Medical Sciences, University of Sydney, Sydney, New South Wales, Australia; b Climate Change Cluster (C3), Faculty of Science, University of Technology Sydney, New South Wales, Australia; Cornell University

**Keywords:** diatom, evolution, microalgae, virome, virosphere, metagenomics

## Abstract

Unicellular microalgae are of immense ecological importance with growing commercial potential in industries such as renewable energy, food, and pharmacology. Viral infections can have a profound impact on the growth and evolution of their hosts. However, very little is known of the diversity within, and the effect of, unicellular microalgal RNA viruses. In addition, identifying RNA viruses in these organisms that could have originated more than a billion years ago constitutes a robust data set to dissect molecular events and address fundamental questions in virus evolution. We assessed the diversity of RNA viruses in eight microalgal cultures, including representatives from the diatom, eustigmatophyte, dinoflagellate, red algae, and euglenid groups. Using metatranscriptomic sequencing combined with bioinformatic approaches optimized to detect highly divergent RNA viruses, we identified 10 RNA virus sequences, with nine constituting new viral species. Most of the newly identified RNA viruses belonged to the double-stranded *Totiviridae*, *Endornaviridae*, and *Partitiviridae*, greatly expanding the reported host range for these families. Two new species belonging to the single-stranded RNA viral clade *Marnaviridae*, commonly associated with microalgal hosts, were also identified. This study highlights that a substantial diversity of RNA viruses likely exists undetected within the unicellular microalgae. It also highlights the necessity for RNA viral characterization and for investigation of the effects of viral infections on microalgal physiology, biology, and growth, considering their environmental and industrial roles.

**IMPORTANCE** Our knowledge of the diversity of RNA viruses infecting microbial algae—the microalgae—is minimal. However, describing the RNA viruses infecting these organisms is of primary importance at both the ecological and economic scales because of the fundamental roles these organisms play in aquatic environments and their growing value across a range of industrial fields. Using metatranscriptomic sequencing, we aimed to reveal the RNA viruses present in cultures of eight microalgae species belonging to the diatom, dinoflagellate, eustigmatophyte, rhodophyte, and euglena major clades of algae. Accordingly, we identified 10 new divergent RNA virus species belonging to RNA virus families as diverse as the double-stranded *Totiviridae*, *Endornaviridae*, and *Partitiviridae* and the single-stranded *Marnaviridae*. By expanding the known diversity of RNA viruses infecting unicellular eukaryotes, this study contributes to a better understanding of the early evolution of the virosphere and will inform the use of microalgae in industrial applications.

## INTRODUCTION

Viruses are often considered the most ancient “life forms” (i.e., replicatory agents). As studies of the viromes of increasingly diverse taxa proceed, the more their remarkable ubiquity, diversity, and abundance becomes apparent (1). RNA viruses are by far the most abundant microorganisms in marine systems (2) and play fundamental roles in these environments by infecting and regulating phytoplankton populations (2). RNA viruses that infect unicellular photosynthetic microalgae are also of primary importance for marine resource management due to the significant ecotoxicological effect of some microalgal hosts, including abundant dinoflagellate species (3). There is also growing awareness of the value of microalgal cultures for biofuels, pharmacology, water treatment, food, and the aquacultural industries (4–7). Indeed, the intensive commercial cultivation and production of microalgae populations could be seriously affected by viral disease outbreaks (8). Accordingly, an extensive description of the RNA virus diversity in unicellular microalgae is of importance to better understand their role and impact on natural microalgal populations and in anticipating the consequences of industrial cultivation.

Knowledge of the RNA virosphere in overlooked eukaryotic lineages that evolved billions of years ago—such as the microalgae—could greatly enhance our understanding of the earliest events in RNA virus evolution. With barely 100 species of RNA viruses reported since the first isolation of a microalgae-infecting RNA virus in 2003 (9), our current knowledge of RNA viruses infecting microalgae is limited, representing less than 0.5% of the RNA viruses for which hosts have been formally established (10). This lack of knowledge most likely reflects the historical focus on viruses that cause disease in humans and bioresources (domestic animals, animal and insect vector, plants) rather than those infecting microbial eukaryotes.

The study of global viromes has been revolutionized by metagenomics. By avoiding cultivation limitations and paving the way for the exploration of very diverse environments (soil, water, etc.), the metagenomic era has multiplied the number of RNA viruses described by many thousands (10–13). This is evident in the field of “phycovirology” (the study of algal viruses), for which recent studies investigating RNA viruses using metagenomic approaches have revealed a high diversity and prevalence of RNA viruses in several microalgae lineages (11, 14–21). While the positive-sense single-strand (ss+) picorna-like *Marnaviridae* are the best described family of microalgae-infecting viruses (11, 22, 23), metagenomic studies continue to expand the diversity of microalgal viruses, including identification of the double-strand RNA (dsRNA) viruses from the orders *Ghabrivirales (Totiviridae-like), Durnavirales (Partitiviridae-like), and Martellivirales (Endornaviridae-like)* (19, 20, 24–26), as well as ss+ RNA viruses from the *Sobelivirales (Alvernaviridae), Nodamuvirales (Nodaviridae), Wolframvirales (Narnaviridae)*, and *Cryppavirales (Mitoviridae)* phyla (19, 20, 24, 27, 28). To date, the majority of the microalgal hosts documented to contain RNA viruses are from the Bacillariophyta (diatom) and Dinoflagellata (dinoflagellate) lineages. However, some viruses have been reported from other stramenopile hosts (such as Phaeophytes, Raphidophytes, and Xanthophytes) (9, 20, 28–30) and in some other major groups of microalgae such as the *Rhizaria* (9, 20), Chlorophyta (19, 31), Rhodophyta (20, 25, 26), and, more recently, Haptophyta (20).

To increase our understanding of the RNA virosphere in microalgae, we assessed the diversity of RNA viruses in eight microalgal species, covering the major groups of stramenopiles, including eustigmatophytes (Nannochloropsis oceanica and Nannochloropsis oculata) and diatoms (Thalassiosira weissflogii), alveolates including dinoflagellates (Prorocentrum cf. *balticum*, Prorocentrum lima, Gambierdiscus carpenteri), red algae (Rhodella maculata), and euglenid (Euglena gracilis). By using a “culture-based” metatranscriptomic approach, we combined the power of unbiased detection of ultralarge-scale RNA sequencing with the use of monoorganism culture to assist in associating the viruses identified to their specific algae hosts. Given the high levels of sequence diversity observed in many RNA viruses, we paid particular attention to identifying divergent virus-like sequences.

## RESULTS AND DISCUSSION

**FIG 1:**
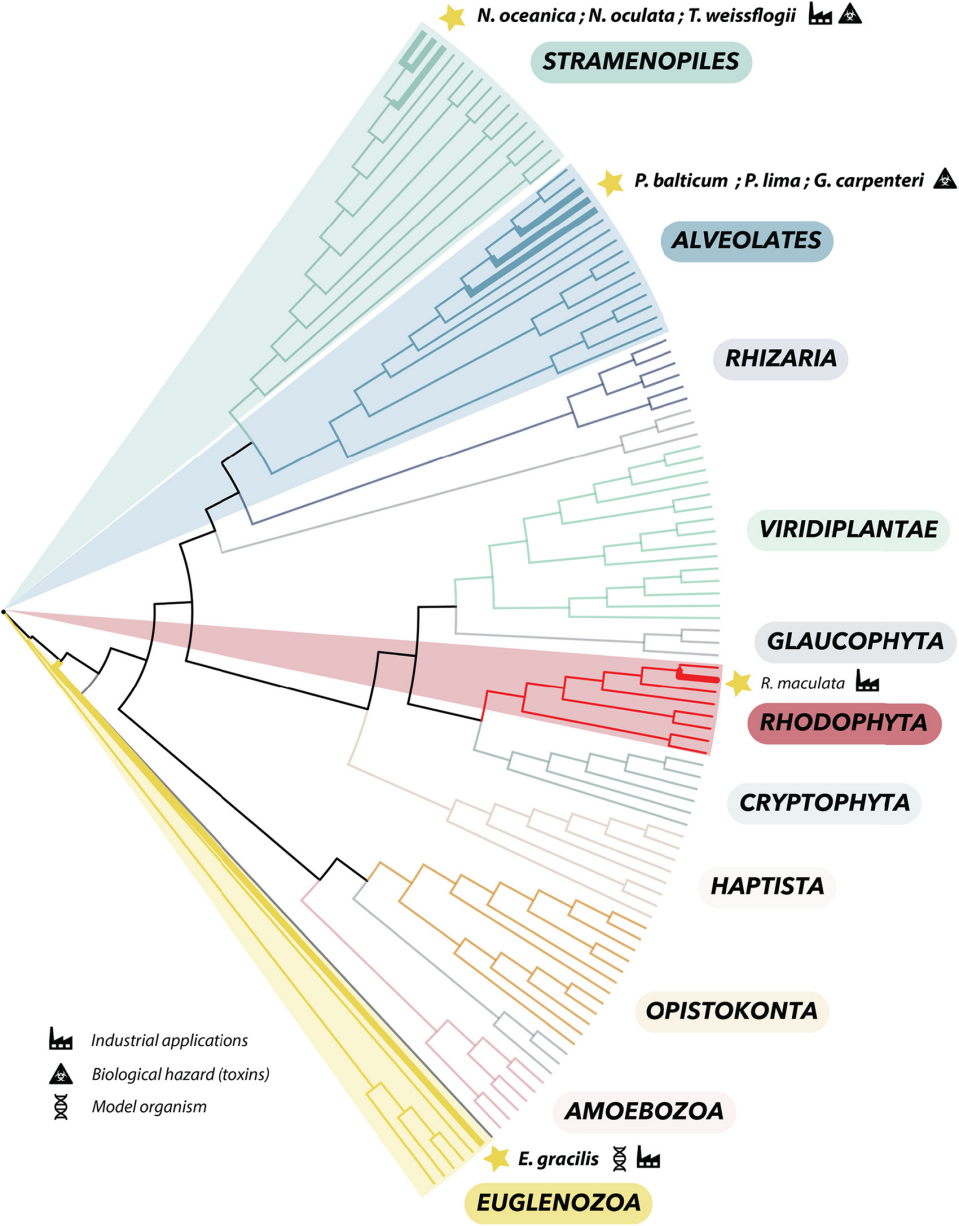
Phylogenetic position of the microalgal species used in this study within the global eukaryote phylogeny. Microalgal species names are indicated in italics, and their main applications (industrial, biological hazard [toxins], and model organisms) are specified by icons. Species for which no RNA viruses were reported prior to this study are indicated in bold. The eukaryote cladogram was based on data from reference 56.

We searched for RNA virus sequences associated with cultures of eight unicellular microalgal species, representing four major algal groups: stramenopiles, alveolates, rhodophytes, and euglenozoa (Fig. 1). Following total RNA extraction from each microalgal culture, metatranscriptomic sequencing was used to obtain deep transcriptomes. The corresponding RNAs and data yields for each microalgal sample/library are detailed in Table 1 and Fig. S1. By combining a standard metagenomic bioinformatic pipeline with the protein hidden Markov model (HMM) profile and structural comparison developed in the RNA-dependent RNA polymerase (RdRp) scan (32), we were able to identify 10 new viral-like sequences (Table 2). With the exception of the unicellular red algae R. maculata, recently associated with the Despoena mito-like virus (20), these represent the first reports of viruses in each microalgal species investigated (Fig. 1). The 10 viral sequences found in this study were compared to the genomic sequences of the corresponding algal host whenever possible (Table S1). Accordingly, 9 of the 10 viral sequences identified were not found in the host genome and therefore were treated as exogenous viruses (Table 2). In contrast, the viral signal detected from E. gracilis using HMM-based approach was identical to Euglena genome sequences (Table 2) and hence likely corresponds to an endogenous viral element (EVE; see below).

**TABLE 1:** Total RNA extractions and RNA-seq results^*a*^

Algae species	Total RNA quantity (ng)	Sequencing data yield (Gb)
Nannochloropsis oceanica	20	27.81
Nannochloropsis oculata	30	52.54
Thalassiosira weissflogii	105	29.39
Prorocentrum lima	700	25.16
Prorocentrum cf. balticum	7,500	40.80
Gambierdiscus carpenteri	630	51.80
Rhodella maculata	120	24.94
Euglena gracilis	5,700	24.90

a RNA-seq, transcriptome sequencing.

**TABLE 2:** New viruses and endogenous viral elements found in this study^*a*^

Virus name	Host (lineage)	RdRp phylum/order related	RT-PCR validated? (virus/host)	Coverage (quality/nb reads)	Length	Full-length vs. partial	Exogenous vs. EVE
Taphios ghabri-like virus 1	Nannochloropsis oceanica (Eustigmatophyte)	Duplornaviricota/Ghabrivirales	Yes/Yes	Good/13,769	4,876	Likely complete	Exogenous
Taphios ghabri-like virus 1	Thalassiosira weissflogii (Eustigmatophyte)	Duplornaviricota/Ghabrivirales	Yes/Yes	Good/876	4,835	Likely complete	Exogenous
Triopas ghabri -like virus 1	Prorocentrum cf. balticum (Dinophyceae)	Duplornaviricota/Ghabrivirales	No/Yes	Average/70	1,425	Partial	Exogenous
Diktys durna-like virus 1	Prorocentrum lima (Dinophyceae)	Pisuviricota/Durnavirales–Partitivirus	Yes/Yes	Good/24,826	1,826	Partial (one segment missing?)	Exogenous
Orion durna-like virus 1	Gambierdiscus carpenteri (Dinophyceae)	Pisuviricota/Durnavirales	Yes/Yes	Good/1,198	2,077	Partial (one segment missing?)	Exogenous
Almopos endorna-like virus 1	Gambierdiscus carpenteri (Dinophyceae)	Kitrinoviricota/Martellivirales	Yes/Yes	Good/54,823	21,494	Likely complete	Exogenous
Althepos endorna-like virus 1	Gambierdiscus carpenteri (Dinophyceae)	Kitrinoviricota/Martellivirales	Yes/Yes	Good/460	4,825	Likely complete	Exogenous
Phineus pisuviri-like virus 1	Rhodella maculata (Rhodophyta)	Pisuviricota/Picornavirales	Yes/Yes	Good/14,972	6,398	Likely complete	Exogenous
Megareus marna-like virus 1	Nannochloropsis oculata (Stramenopiles)	Pisuviricota/Picornavirales	No/No	Good/425	1,222	Partial	Exogenous
Minyas marna-like virus 1	Nannochloropsis oculata (Stramenopiles)	Pisuviricota/Picornavirales	No/No	Good/472	1,130	Partial	Exogenous
Pisuviri-like signal	Euglena gracilis (Euglenozoa)	Pisuviricota/uncertain placement	Yes/Yes	Good/1,531	1,484	EVE	EVE

^*a*^Full-length versus partial information was hypothesized from genomic length and organization. EVE, endogenous viral element; RdRp, RNA-dependent RNA polymerase; RT-PCR, reverse transcription PCR.

**FIG 2:**
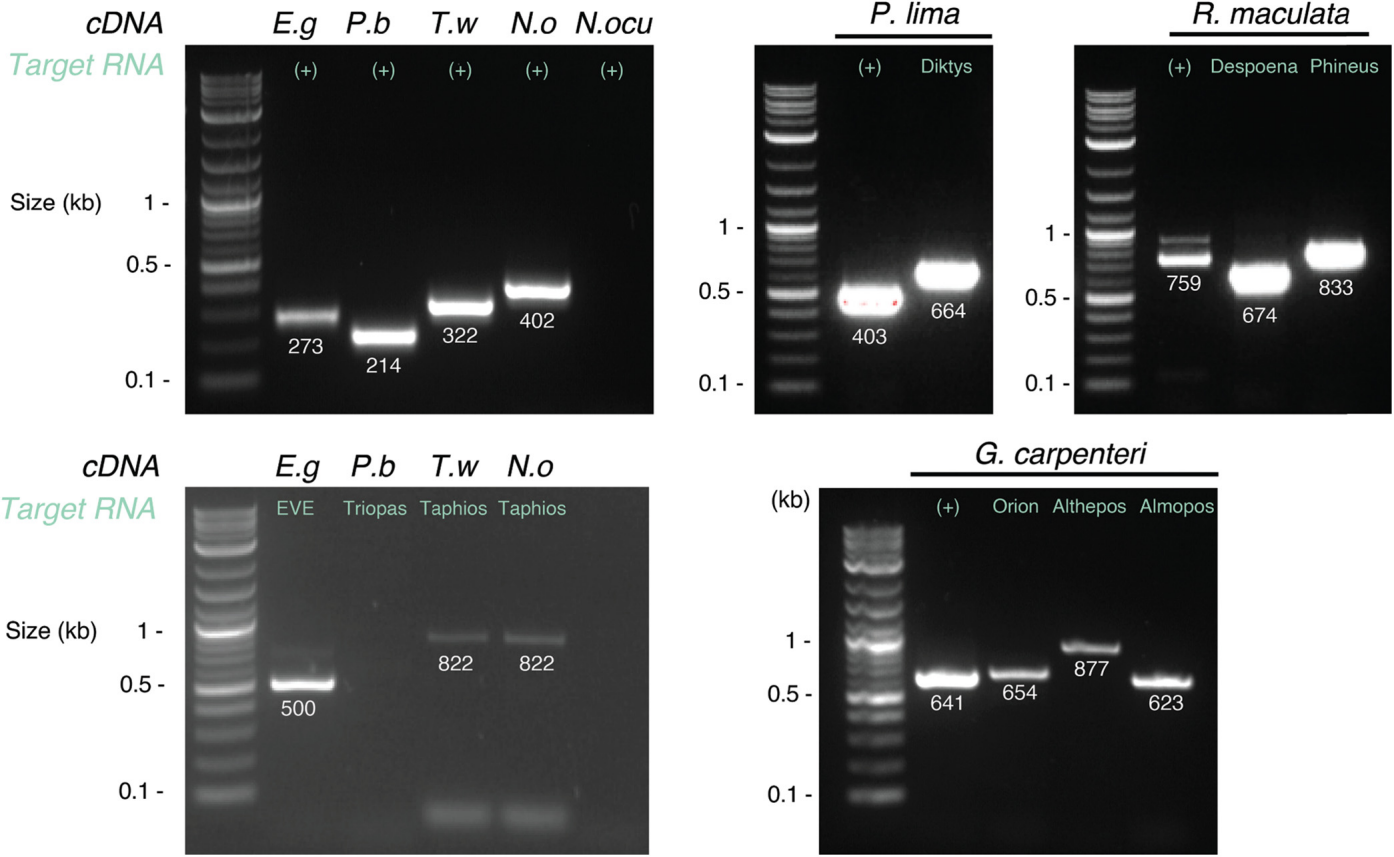
Reverse transcription (RT)-PCR confirmation of novel viral signals identified in this study. The expected lengths of each PCR product are indicated below each band. The RNA sequences targeted for each reaction are indicated in green. The corresponding RNA samples are indicated on top of each well. E.g, Euglena gracilis; P.b, *Prorocentrum* cf. *balticum*; T.w, Thalassiosira weissflogii; N.o, Nannochloropsis oceanica; N. ocu, Nannochloropsis oculata; (+), host gene tested.

To eliminate contamination during the library preparation or sequencing, we tested the presence of all viruses in total RNA samples using reverse transcription (RT)-PCR. This resulted in the detection of 8 of the 11 virus/samples tested (Table 2 and Fig. 2). Triopas ghabri-like virus 1 could not be detected in Prorocentrum cf. *balticum* RNAs (Fig. 2), likely because of the very low abundance of this viral contig (Table 2). Megareus marna-like virus 1 and Minyas marna-like virus 1, both associated with the N. oculata sample, similarly could not be confirmed using RT-PCR. In addition, the positive control used to target the N. oculata internal transcribed spacer (ITS) sequence did not return any PCR signal (Fig. 2). Hence, the meager quantity of total RNA extracted from N. oculata cultures (Table 1) may explain the difficulty in validating both host gene and associated viruses using RT-PCR. As a control, all the final viral genomes sequences were cross-checked with every transcriptome sequencing (RNA-seq) library. None of the viral sequences found in this study were identified in the other samples, with the exception of Toti#2, which was present in the N. oceanica and T. weissflogii libraries.

### Placement of the newly identified microalgal viruses within global RNA virus diversity.

**FIG 3:**
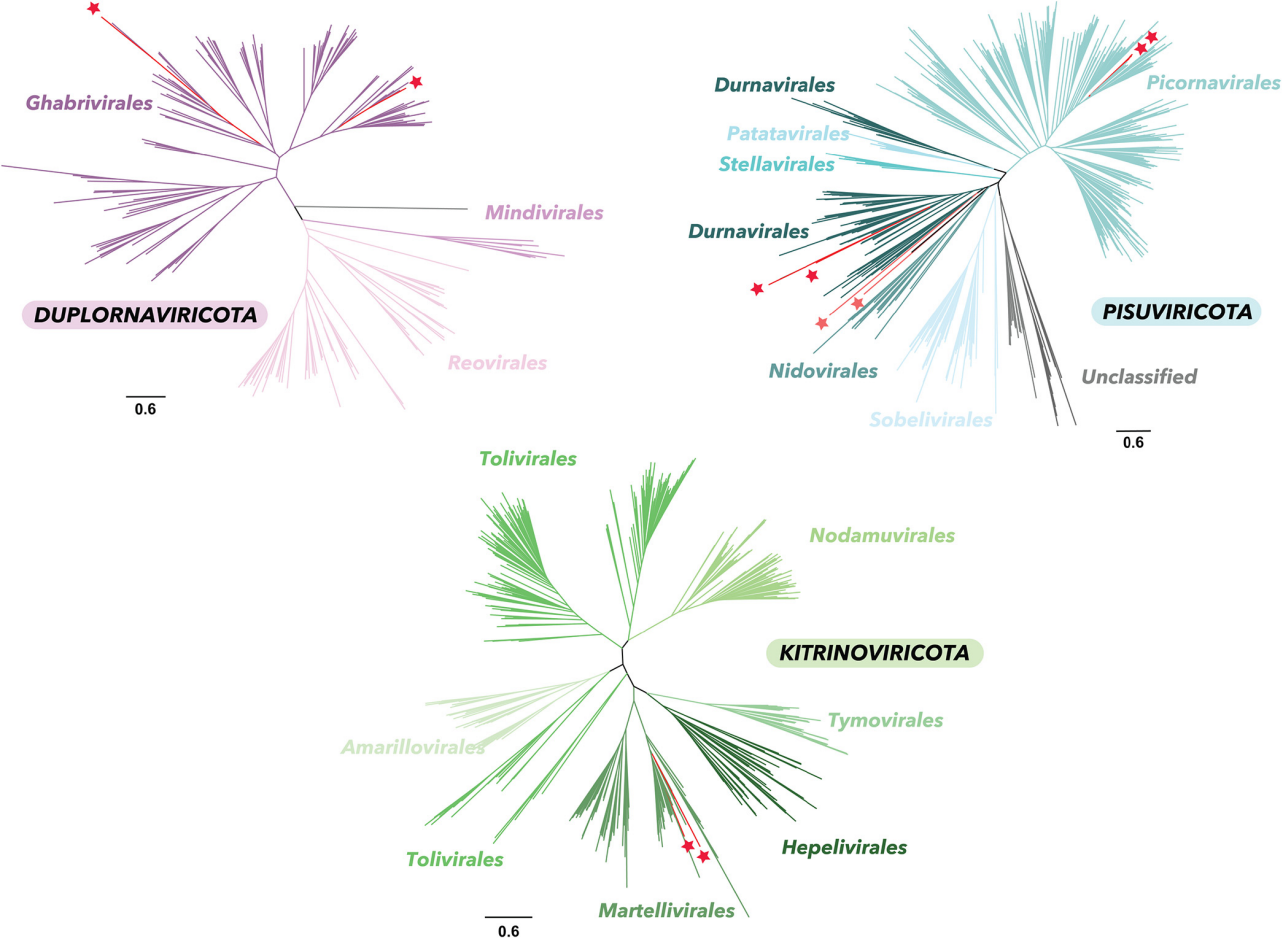
Phylogenetic placement of the newly identified viruses within the diversity of Riboviria phyla. In these unrooted ML trees, red stars indicate the viruses newly identified. Light red stars represent RdRp-like sequences obtained using the hidden Markov model (HMM)-based RdRp-scan method. Bars represent the number of amino acid substitutions per site.

To characterize the newly identified viruses, we first used phylogenetic analysis to place the new viral sequences within the diversity of viral RNA-dependent RNA polymerase (RdRp) sequences at the phylum level using the recently developed RdRp-scan resource (32). These large-scale phylogenies show that the viral sequences identified fell in diverse topological positions among those RNA viruses identified to date, with two belonging to the *Duplornaviricota* viruses, two falling into the *Kitrinoviricota* viruses, and six sharing homologies at amino acid level with *Pisuviricota viruses* (Fig. 3).

We then conducted additional phylogenetic analyses focusing on the viral subclades that contained the 10 newly identified sequences. These comprised the *Ghabrivirales, Endornaviridae, Durnavirales, and Marnaviridae* lineages and are described below.

### New microalgae-infecting viruses suggest a TSAR-infecting Totiviridae genus.

**FIG 4:**
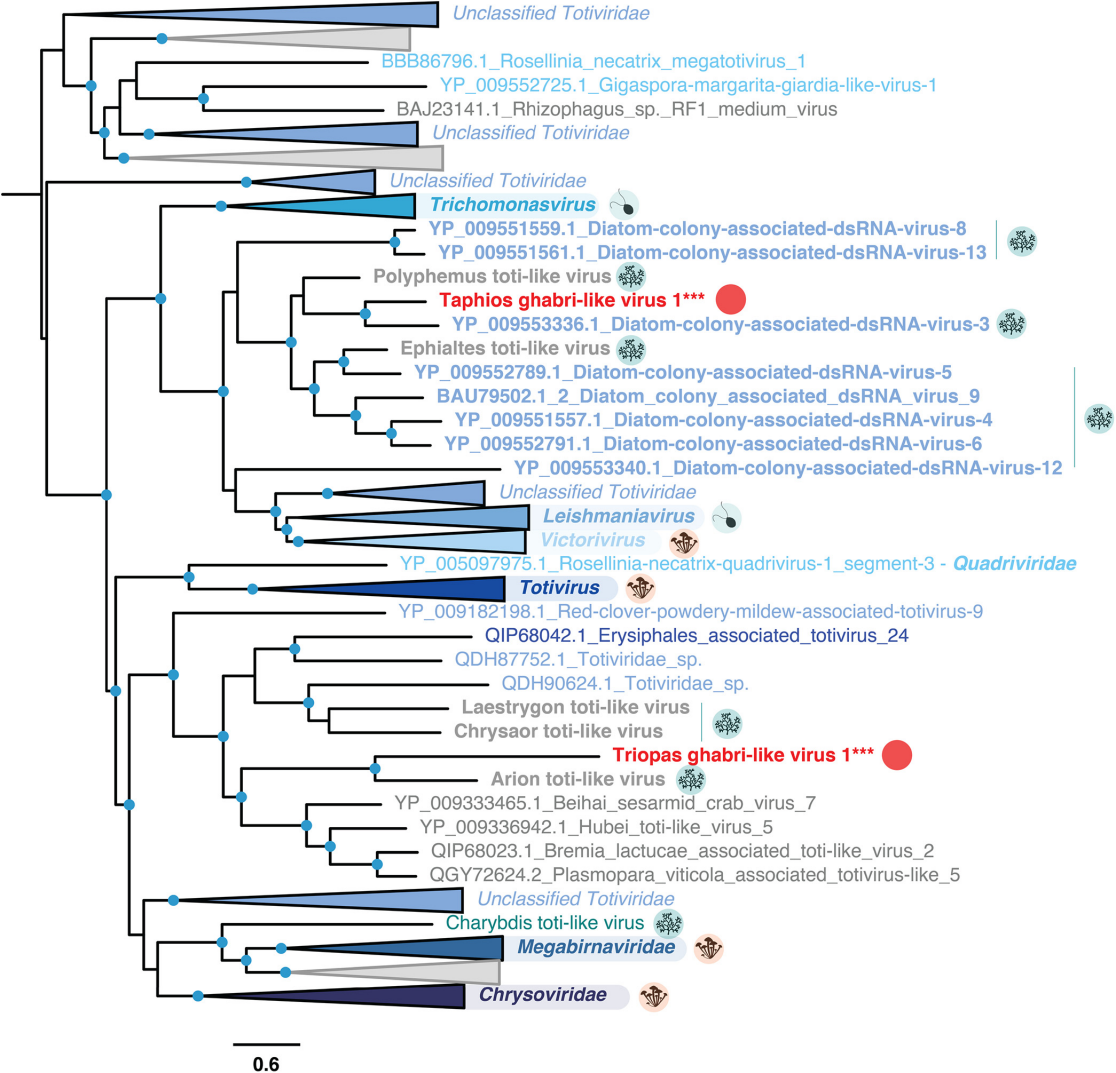
Phylogeny of the Ghabrivirales. Sequences in gray denote previously unclassified viruses, while those in bold refer to microalgae-associated viruses. Host lineages are indicated in circles to the right of major viral clade labels and correspond to fungi (orange), protozoa (light blue), and microalgae (blue). The new viral sequences identified in this study are indicated with red circles. The tree is mid-point rooted, and confident nodes (with SH-alrt likelihood ratio test values ≥80%) are represented as circles. The bar represents the number of amino acid substitutions per site.

Among the 10 viral sequences identified in this study, two were related to *Totiviridae*-like viruses (Table 2 and Fig. 3). Triopas ghabri-like virus 1, identified in the dinoflagellate Prorocentrum cf. *balticum*, forms a clade with the Arion toti-like virus identified in the dinoflagellate Pyrodinium bahamense (20). Together, these two viruses group with those previously reported in microalgae and oomycete hosts (Fig. 4).

**FIG 5:**
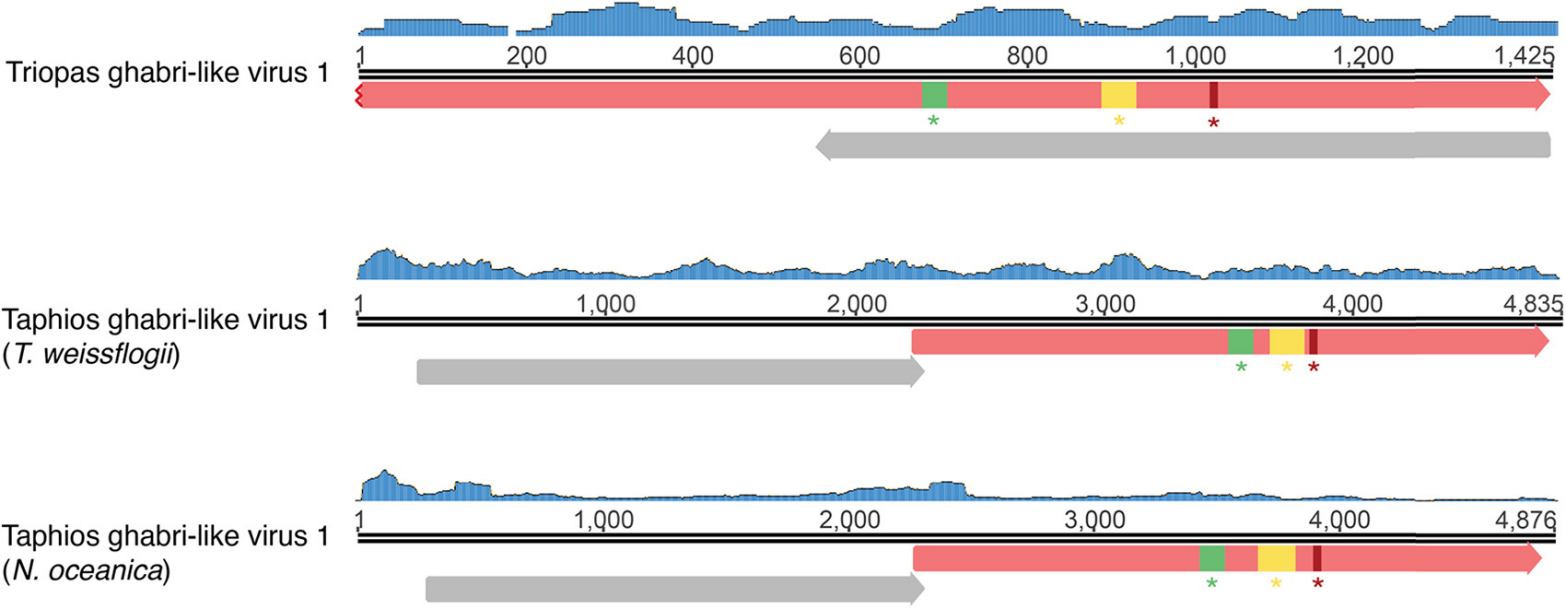
Genome organization of the Ghabri-like viruses identified in this study. Read coverage of each genome is represented as a blue histogram. The open reading frames (ORFs) were predicted using standard genetic codes, and their directions are represented as arrows. ORFs encoding RdRp-like signals and hypothetical functions are indicated in red and gray, respectively. A, B, and C RdRp motifs are indicated in green, yellow, and red boxes, respectively.

The short length of the RdRp-encoding segment identified here—1.4 kb—suggests that the genome of Triopas ghabri-like virus 1 is partial (Fig. 5). While this limits the discussion of genomic attributes, an additional open reading frame (ORF) in reverse orientation and without any known function associated was predicted using the standard genetic code. Such a use of antisense ORF would constitute an original feature in the *Totiviridae*. Triopas ghabri-like virus 1-associated RNAs were found at very low abundance in the Prorocentrum cf. *balticum* sample and could not be confirmed experimentally by RT-PCR (Table 2 and Fig. 2). Although this viral sequence requires additional validation, it supports previous suggestions of dinoflagellate-infecting *Totiviridae* (20) and constitutes further evidence for recognizing a new genus infecting the Telonemid, Stramenopile, Alveolate, and *Rhizaria* supergroup (TSAR) within the *Totiviridae* (20).

A second Toti-like virus, Taphios ghabri-like virus 1, was found in the eustigmatophyte N. oceanica and the diatom T. weissflogii. It forms a clade with the algae-associated Polyphemus and Ephialtes toti-like viruses, both previously identified in Astrosyne radiata (diatom) samples (20). They also form a sister clade to the Trichomonasvirus, Victorivirus, and Leishmaniavirus genera, infecting protozoan parasites and fungi (33–35). To consolidate the host-virus relationship and potentially elongate the genomic sequence, we screened for the presence of the newly described viruses in additional host transcriptomes available in the Sequence Read Archive (SRA) (Table S2). Accordingly, Taphios ghabri-like virus 1 sequences were observed in one transcriptome (SRR12347810) of the diatom Phaeodactylum tricornutum, with only eight single-nucleotide polymorphisms (SNP) reported at the genome level.

The total length of the Taphios ghabri-like virus 1 sequence, at 4.8 kb, is in the range of other *Totiviridae* and, along with the read coverage profile, suggests that the full-length genome has been obtained (Fig. 5). The organization of the Taphios ghabri-like virus 1 genome into two overlapping ORFs, probably translated with a +1 ribosomal frameshift, corresponds to the genomic features commonly observed among the *Totiviridae*. The first ORF likely encodes a coat protein, while no annotations could be retrieved from InterProscan analysis for this ORF (36). We hypothesize from the placement within the *Totiviridae* phylogeny and the similarities in genome organization and length that this virus has a dsRNA genome. Combined, the results from RdRp phylogenies, genome organization, and host range are in accord with establishing a new *Totiviridae* genus infecting diatom and eustigmatophyte hosts.

The observation of *Totiviridae* likely infecting dinoflagellates, diatoms, and eustigmatophyte hosts aligns with the suspected ubiquity of these dsRNA viruses in microalgae (20) and unicellular eukaryotes more generally (37). Notably, the *Totiviridae* have been associated with changes in host fitness and to hyper- or hypovirulence of some of their hosts (38–40). The effects of the newly discovered *Totiviridae* genus on corresponding dinoflagellate, diatom, and eustigmatophyte microalgal cultures require additional investigation and could be of interest considering their potential effects on growth, including that of harmful algal blooms (HABs), and commercial cultivation yields.

### First association of Alphaendornavirus with dinoflagellates.

**FIG 6:**
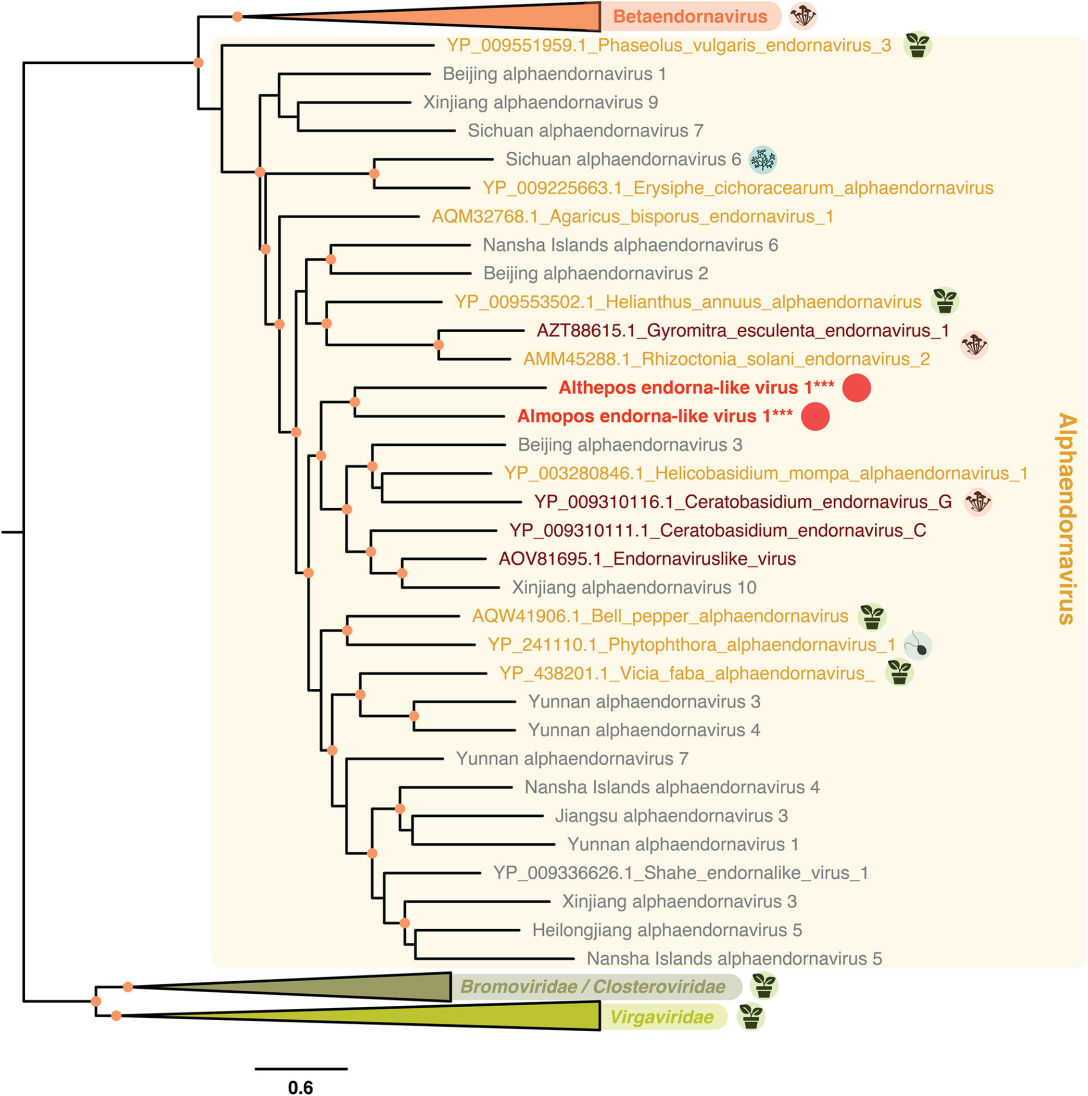
Phylogeny of the Endornaviridae. Sequences in gray denote unclassified viruses and sequences in bold refer to microalgal-associated viruses. Host lineages are indicated in circles to the right of major viral clades and correspond to fungi, land plants, protozoa, and microalgae. The new viral sequences identified in this study are indicated with circles. The Sichuan Alphaendornavirus cluster includes the diatom-associated RNA virus 15, previously reported from diatom-containing samples (18). The tree is mid-point rooted, and confident nodes (with SH-alrt likelihood ratio test values ≥80%) are represented as circles. The bar represents the number of amino acid substitutions per site.

Two of the viruses identified in this study cluster within the *Endornaviridae* family of dsRNA viruses. Specifically, Althepos endorna-like virus 1 and Almopos endorna-like virus 1 (both retrieved from G. carpenteri–Dinophyceae) group with members of the Alphaendornavirus genus, a genus within the *Endornaviridae* previously associated with land plants, fungi, and oomycetes (41) (Fig. 6).

*Endornaviridae* dsRNA genomes are 9.7 to 17.6 kb in length and encode a single polyprotein with a RdRp domain located in the C terminus (37). The genome organization of Almopos endorna-like virus 1 therefore possesses features common to the *Endornaviridae* (Fig. S2), except for its genome size of ~21 kb, which is the longest genome reported to date for this group. In addition to the viral RdRp domain located in the C-terminal region of the Almopos endorna-like virus 1 protein, other protein domains and signatures could be identified that were related to the (+)RNA virus helicase core (IPR027351), the YbiA-like superfamilies (IPR037238), and the UDP-glycosyltransferase/glycogen phosphorylase superfamily (SSF53756) (Fig. S2), similar to previous studies (42–45). It is very likely that other viral proteins and functions are encoded but are too divergent to be identified. The investigation of these additional divergent viral translated products could be of significant importance for both, revealing the evolutionary origins of the *Endornaviridae* (46).

The Althepos endorna-like virus 1 sequence is only 4.8 kb in length and likely represents a partial genome. Additional read mapping using our metagenomic or SRA-based data did not allow the retrieval of the full-length sequence (Fig. S2). Whether Althepos endorna-like virus 1 and Almopos endorna-like virus 1 affect the fitness of their G. carpenteri host remains to be investigated but may have important implications for the management of this potentially harmful species (47). Considering the persistent lifestyle reported for endornaviruses (48) and the high similarities in terms of RdRp sequence and genomic organization, it is likely that Althepos endorna-like virus 1 and Almopos endorna-like virus 1 share the same infectious properties as other members of the genus Alphaendornavirus and might therefore constitute another example of capsid-less persistent viruses associated with protist hosts. Importantly, Althepos endorna-like virus 1 and Almopos endorna-like virus 1 identified from the G. carpenteri culture represent the second microalgal-endornavirus association observed to date (18) and the first report in a dinoflagellate host, strongly suggesting that a microalgal-specific *Endornaviridae* clade may exist.

### A new Partitiviridae genus associated with dinoflagellate hosts.

**FIG 7:**
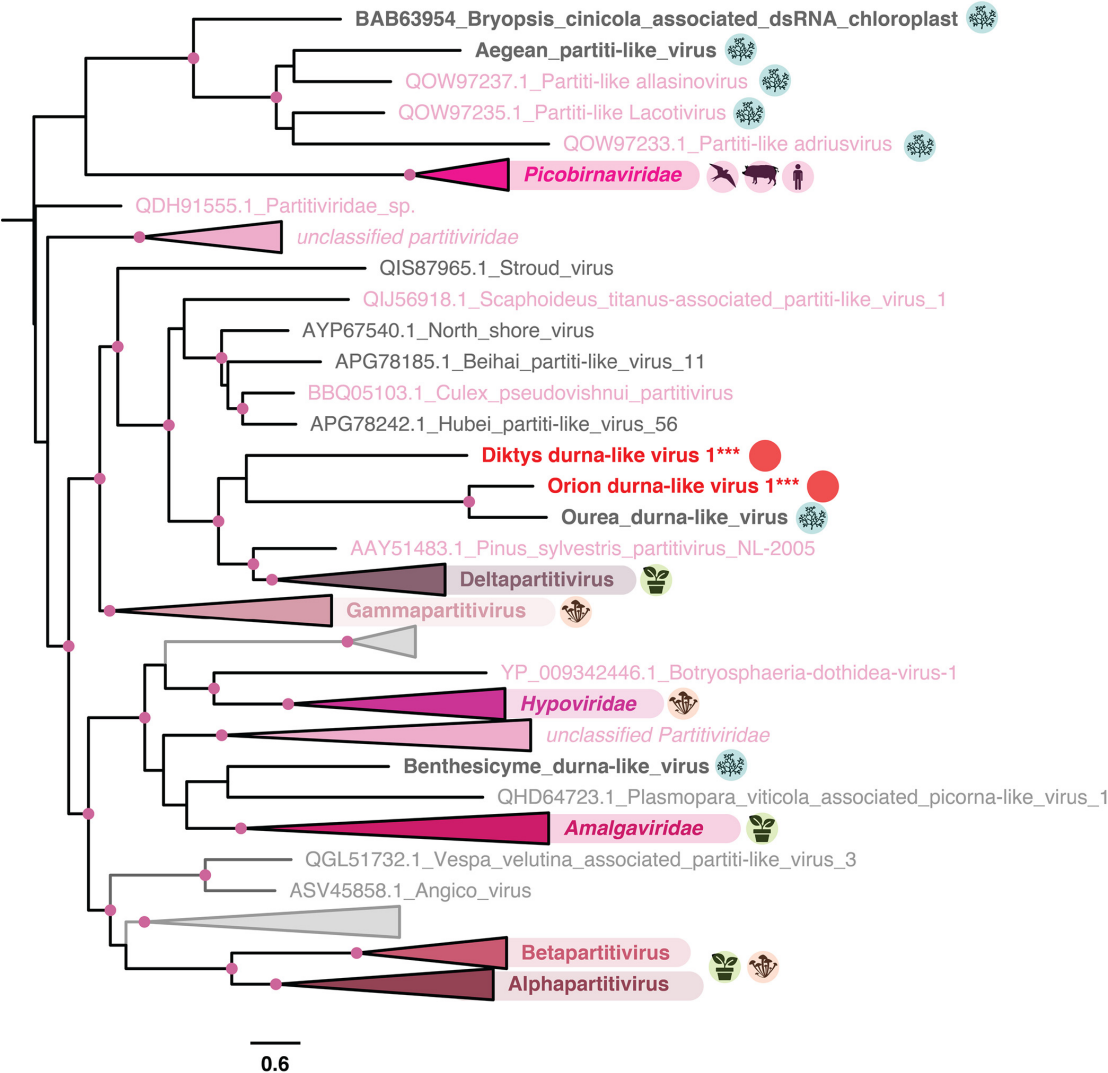
Phylogeny of the Durnavirales. Sequences in gray denote unclassified viruses, while those in bold refer to microalgal-associated viruses. Host lineages are indicated with circles to the right of major viral clades and correspond to metazoa, fungi, land plants, protozoa, and microalgae. The new viral sequences identified in this study are indicated with circles. The tree is mid-point rooted, and confident nodes (with SH-alrt likelihood ratio test values ≥80%) are represented as circles. The bar represents the number of amino acid substitutions per site.

Orion durna-like virus 1 and Diktys durna-like virus 1, observed in P. lima (Dinophyceae) and G. carpenteri (Dinophyceae) cultures, respectively, form a clade with the Ourea durna-like virus previously associated with the dinoflagellate Dinophysis acuminata (20) (Fig. 7). Specifically, they form a sister clade to the genus Deltapartitivirus, belonging to the bi-segmented dsRNA Partitiviridae that infect fungi and plants (49) and recently associated with unicellular algae (20).

Orion durna-like virus 1 and D. durna-like virus 1 genomes (1.8 kb and 2 kb in length, respectively) with a single ORF containing the RdRp domain (Fig. S3). *Partitiviridae* are bisegmented viruses. Considering the placement of Orion durna-like virus 1 and D. durna-like virus 1 within the *Partitiviridae* phylogeny, it is very likely that they comprise a second segment, potentially encoding a coat protein not retrieved in this study due to our RdRp-based retrieval methodology. A complementary comparison of those sequences in the SRA database identified a sequence with 100% sequence identity at the amino acid level to D. durna-like virus 1 from a Gambierdiscus polynesiensis (dinoflagellate) sample (SRR3358210) (Table S2).

Together, these results are compatible with the establishment of a new *Partitiviridae* genus that is specific to dinoflagellates, comprising Orion durna-like virus 1, D. durna-like virus 1, and the previously identified Ourea durna-like virus. This observation expands the host range reported for this family, already comprising plants, fungi, oomycetes, apicomplexan parasites, and green algae (19, 50–53). While most of the *Partitiviridae* do not induce symptoms in their hosts, hypovirulence has been reported in the alpha-, beta-, and gammapartitiviruses (54). Further analysis is required to assess the effects of Orion durna-like virus 1 and D. durna-like virus 1 on their potentially harmful dinoflagellate hosts, G. carpenteri and P. lima (47, 55).

### *Marnaviridae*-like viruses associated with a N. oculata culture.

**FIG 8:**
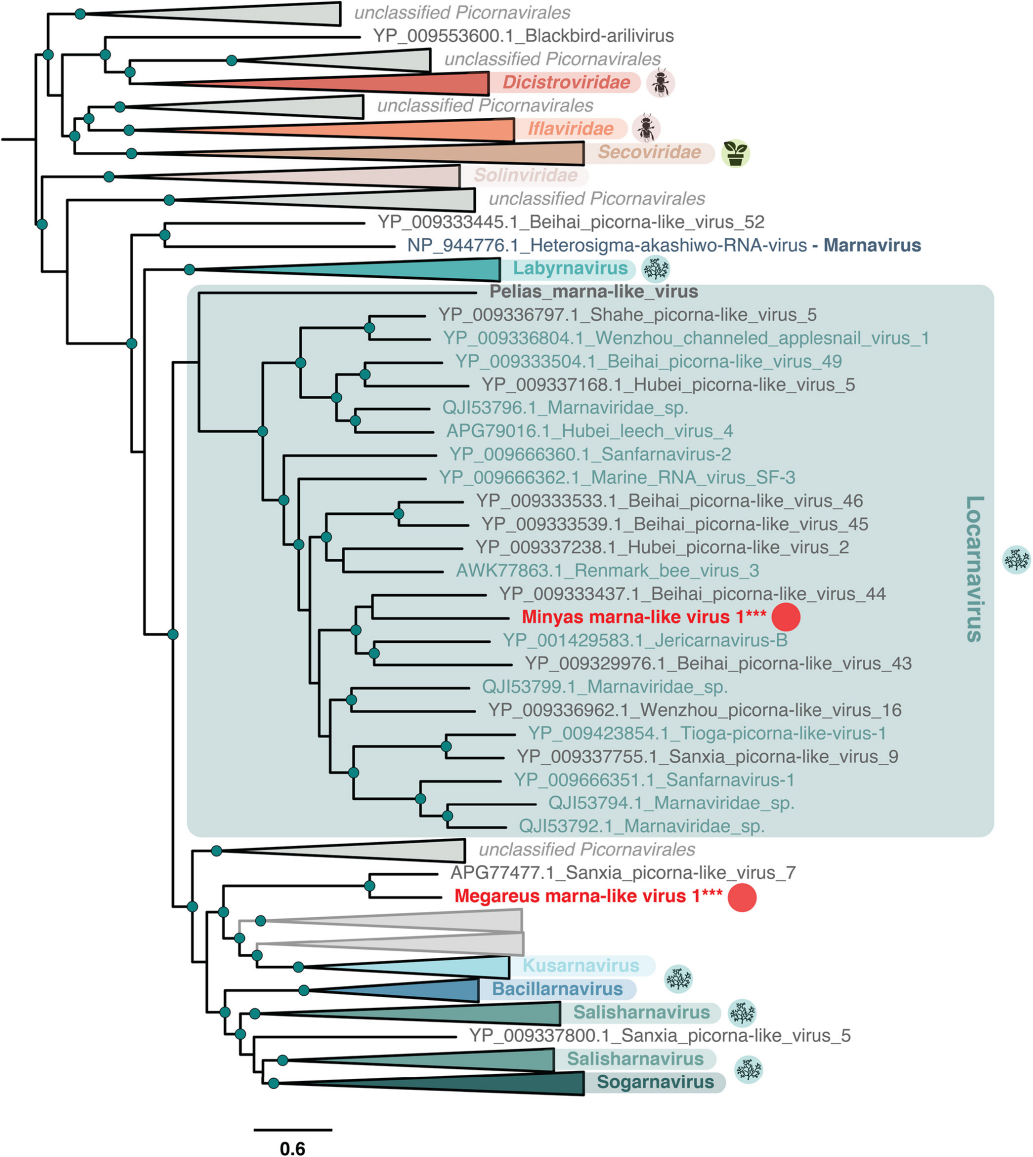
Phylogeny of the Marnaviridae. Sequences in gray denote unclassified viruses, while those in bold refer to algae-associated viruses. Host lineages are indicated in circles to the right of major viral clades and correspond to arthropods, land plants, and microalgae. The new viral sequences identified in this study are indicated with circles. The tree is mid-point rooted, and confident nodes (with SH-alrt likelihood ratio test values ≥80%) are represented as circles. The bar depicts the number of amino acid substitutions per site.

Two newly identified viruses were identified in a N. oculata (a eustigmatophyte) sample and exhibited RdRp sequence similarity with the *Marnaviridae*, a picorna-like family of ss+ RNA viruses that infect unicellular eukaryotes (Fig. 8). With its taxonomy recently reassessed to incorporate viruses from metagenomic studies (22), the *Marnaviridae* are classified into seven genera. Accordingly, Minyas marna-like virus 1 belongs to the genus Locarnavirus that comprises viruses derived from marine environment, mollusc- and fish-based metagenomic studies (Fig. 8).

This identification of Minyas marna-like virus 1 from the N. oculata culture provides compelling evidence for a Locarnavirus directly associated with a unicellular microalga. Along with the previous identification of the Dinophyceae-associated Pelias marna-like virus (20) (Fig. 8), this supports the idea of an extensive host range of locarnaviruses among unicellular microalgae. The second *Marnaviridae*-like virus, Megareus marna-like virus 1, forms a cluster with Sanxia picorna-like virus 7, falling in a position basal to locarna-, kusarna-, bacillarna-, salisharna-, and sogarnaviruses (Fig. 8). It may therefore constitute a new genus of *Marnaviridae* (22).

The short sequences of both Minyas marna-like virus 1 and Megareus marna-like virus 1 and their average read coverages (Table 2 and Fig. S3) strongly suggest that only partial genomes have been recovered. The low quantities of RNA extracted from the N. oculata sample and the corresponding fragmented RNAs likely explain the poor coverage for the corresponding viral contigs and why RT-PCR targeting both viral and host sequences returned negatives (Fig. 2). Additional studies are needed to achieve the genomic and biological characterization of those new *Marnaviridae*-like viruses associated with the eustigmatophyte host N. oculata. In particular, if the two newly reported *Marnaviridae* caused lysis of the biofuel-producing N. oculata cells, this could represent a major concern for industrial-scale production.

### Divergent viruses identified using protein profiles and structural comparisons.

To help identify viruses in basal and divergent microbial eukaryotes, we conducted an approach based on HMM and structural RdRp comparisons, using the newly developed RdRp-scan tool (32). Briefly, ORFs were predicted from each orphan contig and compared to RdRp profiles using hidden Markov models (32). Such a strategy is expected to detect distant homologs sharing less than 30% sequence identity with viral protein sequences currently available in sequence databases. As a result, two additional viral RdRp sequences were identified as distantly homologous to Pisuviricota members (Fig. 3).

Using RdRp-scan HMM profiles, a remote Pisuviricota-like RdRp signal was identified as being associated with E. gracilis. The complementary Phyre2-based homology search returned a strong hit to the picornavirus sicinivirus 3dpol RdRp, validating the Pisuviricota-like signal previously detected. As noted above, comparison with E. gracilis nuclear (GCA_900893395) and mitochondrial (GCA_001638955) revealed strong identities (Table S1). A very close sequence (seven SNPs at the genome level and six nonsynonymous substitutions at the RdRp level) could also be retrieved from the SRA database sample (SRR2294740), corresponding to the mitochondrial genome of E. gracilis. Such a mitochondrial sublocation is also suggested by the ORF found that can be expressed using the Chlorophycean mitochondrial genetic code (Fig. S3). Hence, this Pisuviri-like signal appears to be part of the host genome, likely corresponding to an endogenous viral element. The presence of such EVEs will help identify divergent viruses infecting euglenoid lineages, which are expected to be highly divergent, considering the basal placement of euglenoids within eukaryotic organism diversity (56).

The Phineus pisuviri-like virus 1, identified from R. maculata, was also confidently identified as a remote homolog of Pisuviricota RNA viruses using both RdRp-scan profiles and Phyre2 server. Although its distant and basal position in the RNA virus phylogeny prevents a robust comparison with existing *Riboviria* clades, its genome of 6.4 kb and the associated read coverage suggests the full-length genome was recovered. The genome encodes three ORFs that possess RdRp function at the C terminus (Fig. S3). No functions could be associated with the additional ORFs, and further studies are required to characterize this newly identified protist-infecting virus.

### Additional viruses.

While it does not constitute a novel virus, one contig assembled from the R. maculata was retrieved in very high quantity and identical to the D. mito-like virus (Table 2), previously reported in another R. maculata sample (20). This strongly reinforces the idea of a D. mito-like virus infecting the red algal host and more generally the establishment of a mitovirus subclade able to infect microalgae (20). Finally, our unbiased metagenomic analysis also retrieved additional viruses related to the *Tombusviridae*, with identical sequences identified across several unrelated samples. It is very likely that these sequences result from contamination from kits or water used to extract or prepare RNA and cDNA libraries and were thus discarded from this study.

### Virus-host assumptions based on the composition of microalgal cultures.

**FIG 9:**
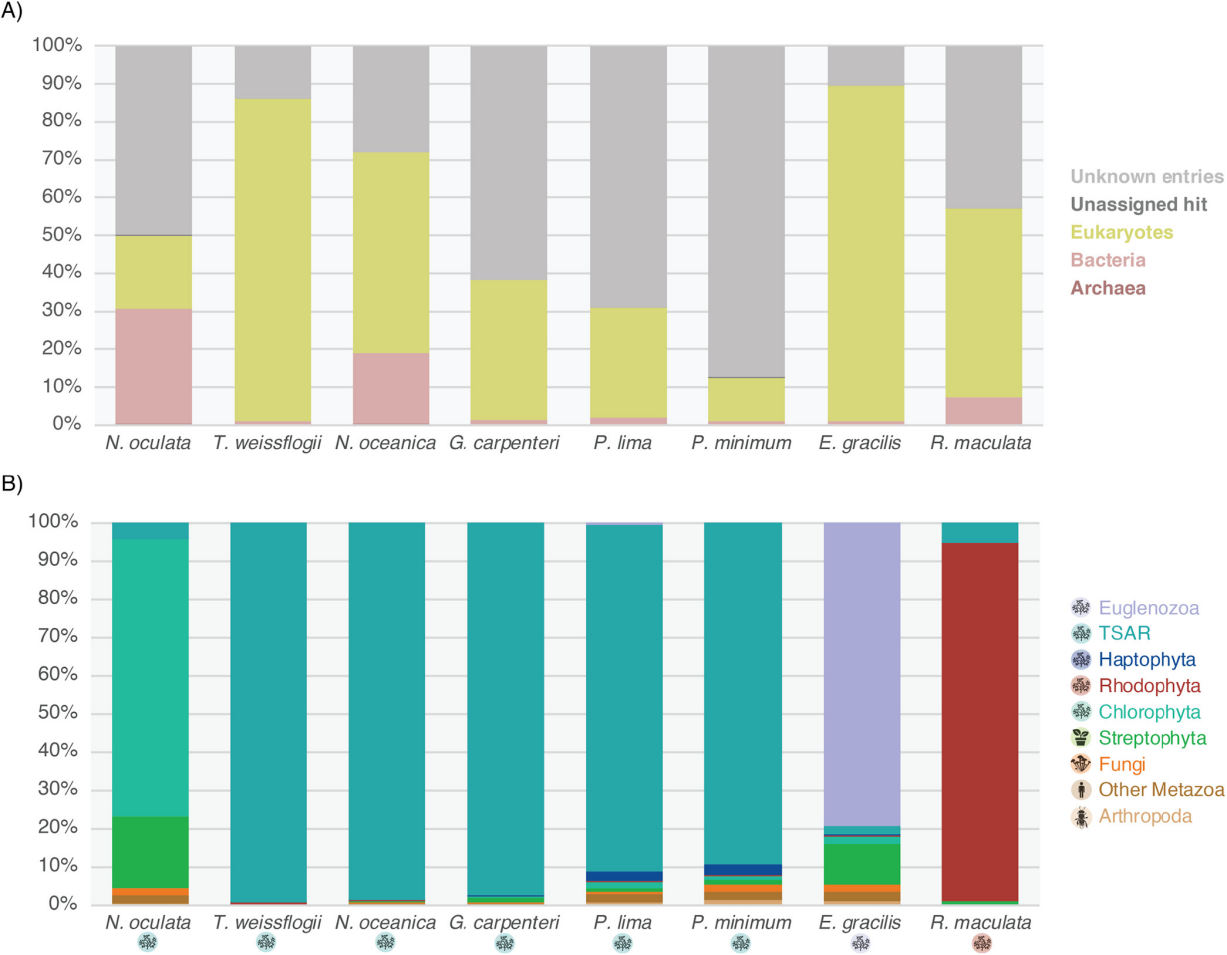
Relative abundance of contigs in microalgae libraries based on their assignment to major cellular organism clades. Contigs were assigned according to the taxonomy of their best BLAST hits. Percentages of each contig were based on the abundance values and correspond to the sum of all contig TPM values belonging to each taxonomy clade. (A) Relative abundance of contigs associated with kingdoms Archaea (dark pink), Bacteria (light pink), and Eukaryota (yellow) using both BLASTn and BLASTx. The abundance of contigs with nt or nr entries lacking a taxonomy assignment are indicated in dark gray, while those without any nt or nr matches detected are indicated in light gray. (B) Relative abundances of contigs associated with major eukaryotic clades using BLASTx. Low abundance clades, counting for less than 0.5% of the total contig abundance, are not represented. TSAR, Telonemids-Stramenopiles-Alveolates-Rhizaria group as defined in reference 56.

The composition of the major kingdoms present in each sample was obtained by comparing contigs to the nt and nr databases. The corresponding proportions and the abundance of contigs without a detectable match in nt or nr are shown in Fig. 9. Bacteria-associated contigs were present within the libraries, especially those from Nannochloropsis and Rhodella, in line with the commonly reported microalgal-bacteria interactions (57). Bacterial and eukaryotic viruses are usually too distantly related to be confounded. The presence of bacterial organisms in the samples is therefore not expected to interfere with our assumption that viruses identified as sharing homology with eukaryotic viruses very likely infect eukaryotic microalgal hosts. The proportion of undetected hits, without any match in the nt and nr databases, is highly variable between libraries, ranging from less than 15% in the T. weissflogii sample to more than 85% in Prorocentrum minimum culture (Fig. 9A). Such high variation likely arises from the lack of microalgal genomic and proteomic sequences in the NCBI nt and nr databases, with genomic sequences available only for half of the microalgae hosts analyzed here (Table S1). Such discrepancies in nucleotide and protein sequence assignment and abundance are further amplified in cases of highly abundant transcripts, such as rRNA, which very likely remain in the sample.

While many unassigned entries in most of the samples analyzed here can limit the formal assignment of viruses to hosts, obtaining a clearer picture of the eukaryotic host sequences present in the sample and their relative abundance can help to discriminate between eukaryotic hosts. Indeed, our cultures were washed several times before RNA extraction. It is therefore likely that the viral sequences identified result from intracellular viral forms rather than extracellular virions circulating in the culture media: hence, we assume that viruses detected in this study are associated with cellular organisms that are also present in the sample. We therefore examined the deep taxonomy of BLASTx eukaryotic-like contigs, as well as the total contig abundance reported for major eukaryotic lineages (Fig. 9B), which helped discriminate potential hosts for the most uncertain assignments. Accordingly, the very low abundance of fungi and land plant-associated sequences in G. carpenteri (Fig. 9B) could constitute additional evidence for a microalgae-infecting endornavirus, even though members of the *Endornaviridae* have been traditionally associated with fungi and land plants (Streptophyta).

In the case of the P. pisuviri-like virus 1 identified from R. maculata, the majority of detectable contigs belong to the corresponding Rhodophyta host taxa, suggesting that this virus is likely associated with a Rhodophyte host rather than fungi or other contaminant organisms. The very large proportion of contigs associated with land plants (Streptophyta) in the N. oculata library might correspond to contamination. However, the unambiguous placement of the corresponding Minyas marna-like virus 1 virus within the well established microalgae-infecting *Marnaviridae* provides a strong argument that this virus is associated with diatoms.

Of note, all of the viruses reported here were identified in healthy algae cultures and therefore assumed to be detrimental to their algal hosts. It will be of considerable interest to characterize the phenotypic effects of such seemingly commensal infections, whether persistent, neutral, or even beneficial, and to understand their underlying molecular basis.

### Conclusions.

Through metatranscriptomic sequencing of total RNA from microalgae cultures we identified 10 new RNA viruses associated with diatom, eustigmatophyte, dinoflagellate, and rhodophyte microalgae. These newly discovered viruses contribute to the establishment of new microalgae-infecting viral clades within the *Totiviridae* and *Partitiviridae*, as well as the enrichment of the positive single-stranded picorna-like family *Marnaviridae*. This study also extended the host range of the dsRNA *endornaviruses* to microalgae, raising questions about how this viral family is able to infect the plant, fungi, and TSAR eukaryotic supergroups. Considering the harmful or commercial value of their hosts, this description of new microalgal viruses paves the way for further studies of the effects of viral infections on host biology and their associated ecological and industrial consequences. Finally, this study highlights the need to reveal the hidden diversity among RNA viruses infecting microalgae and among microbial eukaryotes in general, particularly considering their fundamental and applied importance.

## MATERIALS AND METHODS

### Algae cultures.

Microalgal cultures were maintained on a 12-h:12-h light:dark cycle at 100 μmol m^−2^ s^−1^. Culture media and temperature conditions were specific to each species and were as follows: N. oceanica 24°C, f/2 medium; N. oculata 24°C, f/2 medium; T. weissflogii 20°C, f/2 medium; R. maculata 24°C, L1 medium (minus Si)*;* E. gracilis 20°C, euglena medium; Prorocentrum cf. *balticum* (UTSPH2D4) (47); 20°C K medium-Si; P. lima 25°C modified K medium (58); and G. carpenteri (UTSHI2C4) 25°C modified K medium (47, 59). To harvest each microalgal culture, the cells from 100 to 250 mL were pelleted by centrifugation at 200 × *g* for 4 min and the supernatant discarded. The cells were then resuspended in 5 mL of artificial seawater and centrifuged again at 200 × *g* for 4 min. This wash step was repeated twice more before a final centrifugation step at 1,000 × *g* for 4 min followed by storage at −80°C until RNA extraction.

### Total RNA extraction and sequencing.

Total RNA from the diatom (T. weissflogii) and Euglenozoa (E. gracilis) cultures were extracted using the RNeasy Plus Universal kit (Qiagen), according to the manufacturer’s instructions. Qiazol lysis buffer was then added to frozen pellets, and homogenization was performed by pipetting. Genomic DNA was removed, and RNA was extracted using 1-bromo-3-chloropropane. Supernatants were then transferred to Qiagen columns. After washing the columns, pure RNAs were collected into sterile water strictly following kit instructions.

Total RNA from dinoflagellates (P. lima, Prorocentrum cf. *balticum*, G. carpenteri), the Rhodophyta R. maculata, and the eustigmatophyte (N. oceanica and N. oculata) cultures was extracted using Allprep DNA/RNA kit (Qiagen), following the manufacturer’s instructions. Briefly, frozen cell pellets were supplanted with lysis RLT buffer and cells disrupted using bead beating with 0.5-mm glass beads. An additional step of sample homogenization using QIAshredder (Qiagen) was added during the RNA extraction of R. maculata sample and N. oceanica to reduce the viscosity of eluates. Cell debris was removed using a centrifugation step at high speed, and the supernatants were transferred to Qiagen columns. Total RNA fractions were then purified after several washing steps and eluted according to the kit instructions.

### RNA sequencing.

RNA quality was checked using a TapeStation and individually converted by the Australian Genome Research Facility (AGRF, Melbourne) into non-rRNA RNA-seq libraries using TruSeq Stranded Total RNA with Ribo-Zero Plant (Illumina). Due to the very low RNA yields obtained for N. oculata and N. oceanica, these two libraries were prepared using the SMARTer stranded total RNA-seq kit version 2, Pico Input Mammalian libraries (TaKaRa Bio, Mountain View, CA, USA). The corresponding libraries were sequenced on the NovaSeq platform (Illumina) (paired-end, 150 bp) by the AGRF.

### RNA-seq data preprocessing: read trimming, rRNA depletion, and contig assembly.

Total reads were filtered using Trimmomatic (version 0.36) (60) to remove low-quality and Illumina adapters. To maximize the completeness of the ribosomal RNA (rRNA) depletion performed during library prep, the remaining rRNA reads were removed using the SortmeRNA program (2.1b) (61). Filtered reads were then assembled into contigs using Trinity (version 2.5.1) (62), and the abundances (expected count and TPM) were calculated using RSEM (version 1.3.1) (63).

### Sample taxa composition.

To help determine the taxa composition of each library, all contig sequences were compared to the nonredundant protein database nr from NCBI using Diamond BLASTx (version 2.0.9) (64) and to the nucleotide database nt from NCBI using BLAST (version 2.2.30). The best hits were reported for each contig, and their corresponding taxonomies were analyzed. For each library, contigs were grouped into major eukaryotic taxa and relative abundance determined as the sum of all the TPM (transcripts per million) within each taxon.

### RNA virus identification.

**(i) Sequence-based similarity detection.** RdRp sequences corresponding to RNA viruses (i.e., the *Riboviria*) were first identified by comparing contigs to the nr database using Diamond BLASTX (version 2.0.9; e value < 1e−5) (64). To maximize the detection of RNA viruses, putative virus sequences identified from nr BLASTx, as well as those previously obtained in an algae virus study (20), were used as a database to perform a second round of BLASTx using contig libraries as queries and employing the same parameters as previously. The resulting RNA virus-like sequences were then submitted to the nr database (NCBI), and hits with the best match in cellular organism sequences were treated as false positives and discarded from the analysis.

**(ii) HMM-based homology detection of ORFans.** All orphan contig sequences (i.e., that had no match in the nr database denoted ORFans) were compared to the RdRp HMM profiles of the RdRp-scan resource (32) and using the HMMer3 program (version 3.3) (65).

**(iii) Genome extension, genome coverage, and virus annotation.** To ensure all the RNA virus-like sequences could be identified and in their longest form, additional attempts to assemble contigs were performed using the rnaSPADES (version 3.13.0) (66) and Megahit programs (version 1.2.9) (67). This did not identify additional or longer RNA virus sequences. A manual elongation step was performed on viral candidates using Geneious (version 11.1.4) (68). A virus annotation to identify RdRp motifs was performed using InterProScan (69) and RdRp-scan (32). Genome coverage profiles were obtained by mapping the non-rRNA reads back to each contig sequence using Bowtie2 (version 2.3.3.1) (70) and Samtools (version 1.6) (71). The resulting SAM files were then plotted onto viral genomes using Geneious (version 11.1.4) (68). The overall quality of each genome assembly was finally assessed based on the read coverage homogeneity along the sequence and its decrease at the extremities.

**(iv) SRA mining.** To help retrieve complete genome sequences, assess intraspecies variability, and associate viruses with particular algae hosts, we performed an additional step of Sequence Read Archive (SRA) mining for each of the 10 new viruses identified in this study. For each algae library, we screened the SRA using nucleotide Magic-BLAST (version 1.3.0) (72). When the number of hits exceeded 100, the corresponding SRA reads were mapped to the viral genome using Bowtie2 (version 2.3.3.1) (70) and SAMtools (version 1.6) (71).

**(v) Phylogenetic analysis.** RNA virus phyla-level comparisons were performed using Clustal Omega (version 1.2.4) (73) to directly compare the newly identified sequences to the prebuilt RdRp alignments from the RdRp-scan resource (32). Initial phylogenetic trees were inferred using the maximum-likelihood method available in FastTREE (version 2.1.9; default parameters) (74). Subalignments at the RNA virus order or family scale were then obtained using Clustal Omega (version 1.2.4) (73) and manually checked using Geneious (version 11.1.4) (68). Maximum-likelihood phylogenies of these subalignments were then inferred using the IQ-TREE package (version 2.0-rc1) (75) with the best-fit amino acid substitution model obtained with ModelFinder Plus (76) and using a Shimodaira-Hasegawa approximate-likelihood ratio and 1,000 replicates (-alrt 1,000) to assess nodal support.

### RT-PCR confirmation.

To experimentally confirm viral contigs assembled from RNA-seq data, cDNAs from each of the total RNAs were first obtained using the SuperScript IV reverse transcriptase (Invitrogen). PCRs were then performed on each cDNA sample using corresponding host and virus primers (detailed in Table S3) using the Platinum SuperFi II DNA polymerase (Invitrogen) and following the manufacturer’s instructions.

### Data availability.

Corresponding RNA-seq read files are available on the SRA under BioProject PRJNA867582, with accessions SAMN30215649 to SAMN30215656. The viruses newly identified here are available at GenBank/NCBI under the accessions OP191686 to OP191695.
