# Supplementary material for: Diverse RNA Viruses Associated with Diatom, Eustigmatophyte, Dinoflagellate, and Rhodophyte Microalgae Cultures
Source: J Virol. 2022 Oct 3;96(20):e00783-22. doi: 10.1128/jvi.00783-22 (PMC9599419; doi:10.1128/jvi.00783-22)
Supplement: Supplemental file 1 — Tables SA1 to SA3 and Fig. SA1 to SA3. Download jvi.00783-22-s0001.pdf, PDF file, 0.5 MB [file jvi.00783-22-s0001.pdf]

Appendices

Supplementary Tables

Table A1. Genome BLASTn analysis of the viruses detected in this study. Host algal species in which the closest species are used as surrogates are indicated with a * symbol.

Viral Species	Algae Culture/Library	Algae Species	Genome	Blast
Triopas Ghabri-like Virus 1	*P. cf. balticum*	*P. cf. balticum*	GCA_001652855.1	No hits
Taphios Ghabri-like Virus 1	*N. oceanica*	*N. oceanica*	GCA_004519485.1	No hits
Taphios Ghabri-like Virus 1	*T. weissflogii*	*T. oceanica**	GCA_019693575.1	No hits
Diktys Durna-like Virus 1	*P. lima*	*P. minimum**	GCA_001652855.1	No hits
Orion Durna-like Virus 1	*G. carpenteri*	*G. carpenteri*	Not available	ND
Almopos Endorna-like Virus 1	*G. carpenteri*	*G. carpenteri*	Not available	ND
Althepos Endorna-like Virus 1	*G. carpenteri*	*G. carpenteri*	Not available	ND
Phineus Pisuviri-like Virus 1	*R. maculata*	*R. maculata*	Not available	ND
Megareus Marna-like Virus 1	*N. oculata*	*N. oculata*	GCA_004335455.1	No hits
Minyas Marna-like Virus 1	*N. oculata*	*N. oculata*	GCA_004335455.1	No hits
Pisuviri-like Signal	*E. gracilis*	*E. gracilis*	GCA_900893395.1<br>GCA_001638955 (mitochondrial)	+++

Table A2. Number of SRA accessions screened for the newly identified viruses and their corresponding hits. “SRA nb”: number of SRA libraries used for each algae species; “Hits found”: SRA library accessions for virus positive hits.

Viral species	Library	Algae clade	SRA nb	Hits found
Taphios ghabri-like virus 1	*N. oceanica*	Nannochloropsis	292	None
Taphios ghabri-like virus 1	*T. weissflogii*	Thalassiosira	709	SRR12347810
Triopas ghabri-like virus 1.1	*P. cf. balticum*	Prorocentrum	143	None
Diktys durna-like virus 1	*P. lima*	Prorocentrum	143	None
Orion durna-like virus 1	*G. carpenteri*	Gambierdiscus	86	SRR3358210
Pisuviri-like signal	*E. gracilis*	Euglena	72	SRR2294740
Almopos endorna-like virus 1	*G. carpenteri*	Gambierdiscus	86	None
Althepos endorna-like virus 1	*G. carpenteri*	Gambierdiscus	86	None
Phineus pisuviri-like virus 1	*R. maculata*	Rhodella	3	None
Megareus marna-like virus 1	*N. oculata*	Nannochloropsis	292	None
Minyas marna-like virus 1	*N. oculata*	Nannochloropsis	292	None

Table A3. List of primer sequences used in this study. All PCR reactions were performed at a universal annealing temperature of 60ºC. ITS: Internal Transcribed Spacer. LSU: Ribosomal Large Subunit.

Primer ID	Direction	Sequence (5'-3')	Sequence targeted	length
Euglena-ITS Fw	Forward	TCCTGCCTATCACCCCACA	E. gracilis ITS	273
Euglena-ITS Rev	Reverse	CTACCCCGGTCCCGACTTT		
Pisuviri-like signal Fw	Forward	TGCTGCACCTGCTATGCTT	Pisuviri-like signal	500
Pisuviri-like signal Rev	Reverse	CACGTGTGTCATCCCCACA		
P.minimum_ITS Fw	Forward	CAGTTGGTGAGGCTCTGGG	P. *cf. balticum* ITS	214
P.minimum_ITS_Rev	Reverse	TCGTTGTTCGAGCCGAGAC		
Triopas ghabri-like virus 1	Forward	GCGARATGRTKGTSGARYT	Triopas ghabri-like virus 1	400
Triopas ghabri-like virus 1	Reverse	RTGSCCRATGWRCTGSACG		
ITS1-Fw	Forward	TCCGTAGGTGAACCTGCGG	T.weissflogii ITS	322
Glaucocystis ITS_Rev	Reverse	TCGCTGCGTTCTTCATCGT		
Taphios ghabri-like virus 1	Forward	ACACGGAAAGGTGAGAGCG	Taphios ghabri-like virus 1	822
Taphios ghabri-like virus 1	Reverse	GGGCCAGCATAGCTAGCTC		
N.oculata ITS	Forward	GTGGCCGATTATGGGAGGG	N. oculata ITS	233
Glaucocystis ITS	Reverse	TCGCTGCGTTCTTCATCGT		
ITS1-FWD	Forward	TCCGTAGGTGAACCTGCGG	P. lima ITS	403
P. lima ITS	Reverse	TCAAGGGCCACAGCAAGAC		
Diktys durna-like virus 1	Forward	ACCGCATCTTCGCAACAGA	Diktys durna-like virus 1	664
Diktys durna-like virus 1	Reverse	AGCTTTGCATGCGGGTAGT		
G_carpenteri_LSU	Forward	AGGCTGTGCATGGCTCATT	G. carpenteri LSU	641
G_carpenteri_LSU	Reverse	CAGCCATCCCCAGCAGAAA		
Orion durna-like virus 1	Forward	GCTCGGCTTCAGGTCAGTT	Orion durna-like virus 1	654
Orion durna-like virus 1	Reverse	CACTTGCGTTTTCGGTGGG		
Althepos endorna-like virus 1	Forward	AGTGGCGCACAAGCACTAT	Althepos endorna-like virus 1	877
Althepos endorna-like virus 1	Reverse	CCCCATTTCCAAGCCGTCT		
Almopos endorna-like virus 1	Forward	ACCTTCTTGGCCTGGATGC	Almopos endorna-like virus 1	623
Almopos endorna-like virus 1	Reverse	ATCCTCCTCAGACGGTGCT		
Rhodella_maculata_ITS1	Forward	CGGCCGAGTTCGACTATCC	R. maculata ITS1	759
Rhodella_maculata_ITS1	Reverse	TTCTTTGGGGTAGGCGCTG		
Phineus pisuviri-like virus 1	Forward	TCGGTCCAGATGGCAAACC	Phineus pisuviri-like virus 1	833
Phineus pisuviri-like virus 1	Reverse	CACCGGTGAGCCTTGTGAT		

Supplementary Figures

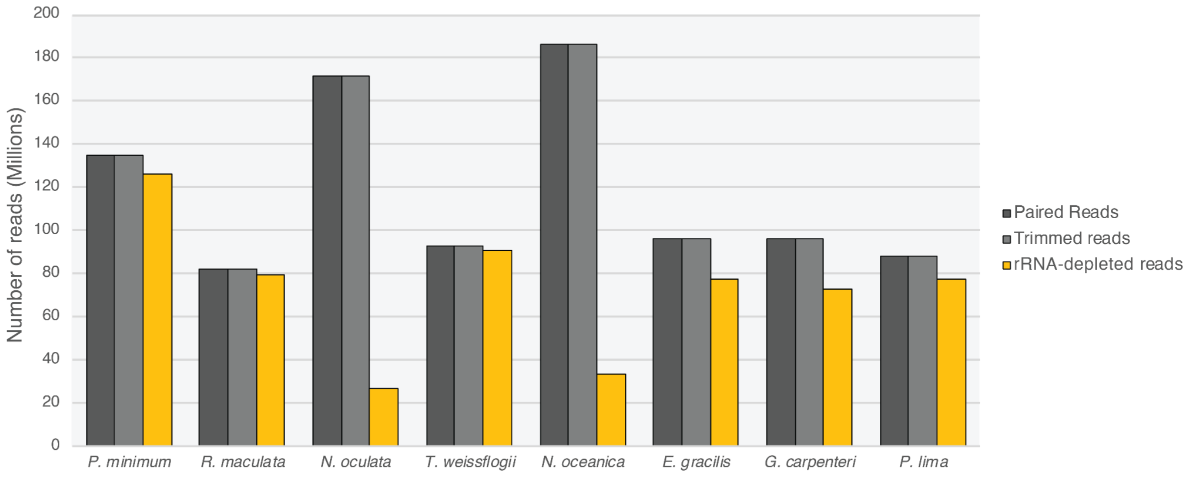

Figure A1. Number of sequencing reads obtained for each algae library. Paired-end reads obtained using the Illumina NovaSeq platform are indicated in dark grey. The final number of paired reads obtained after trimming and rRNA depletion steps are indicated in light grey and yellow, respectively.

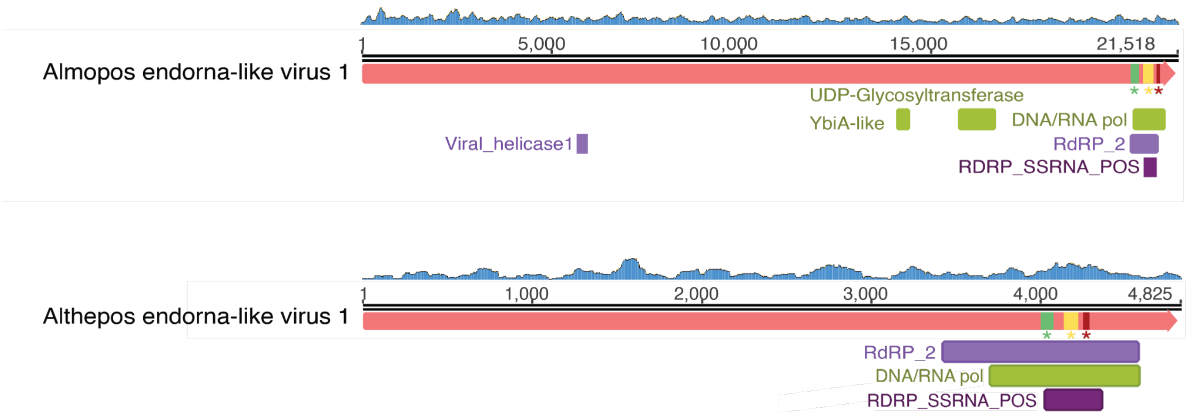

Figure A2. Genome organisation of the Endorna-like viruses identified in this study. Read coverage of each genome is represented as a blue histogram. ORFs were predicted using standard genetic codes and their directions represented as arrows. ORFs encoding RdRp-like signals and hypothetical functions are indicated in red and grey respectively. Light purple, green and dark purple boxes indicate PROSITE, SUPERFAMILY and PFAM predicted domains, respectively. A, B and C RdRp motifs are indicated in green, yellow and red boxes, respectively.

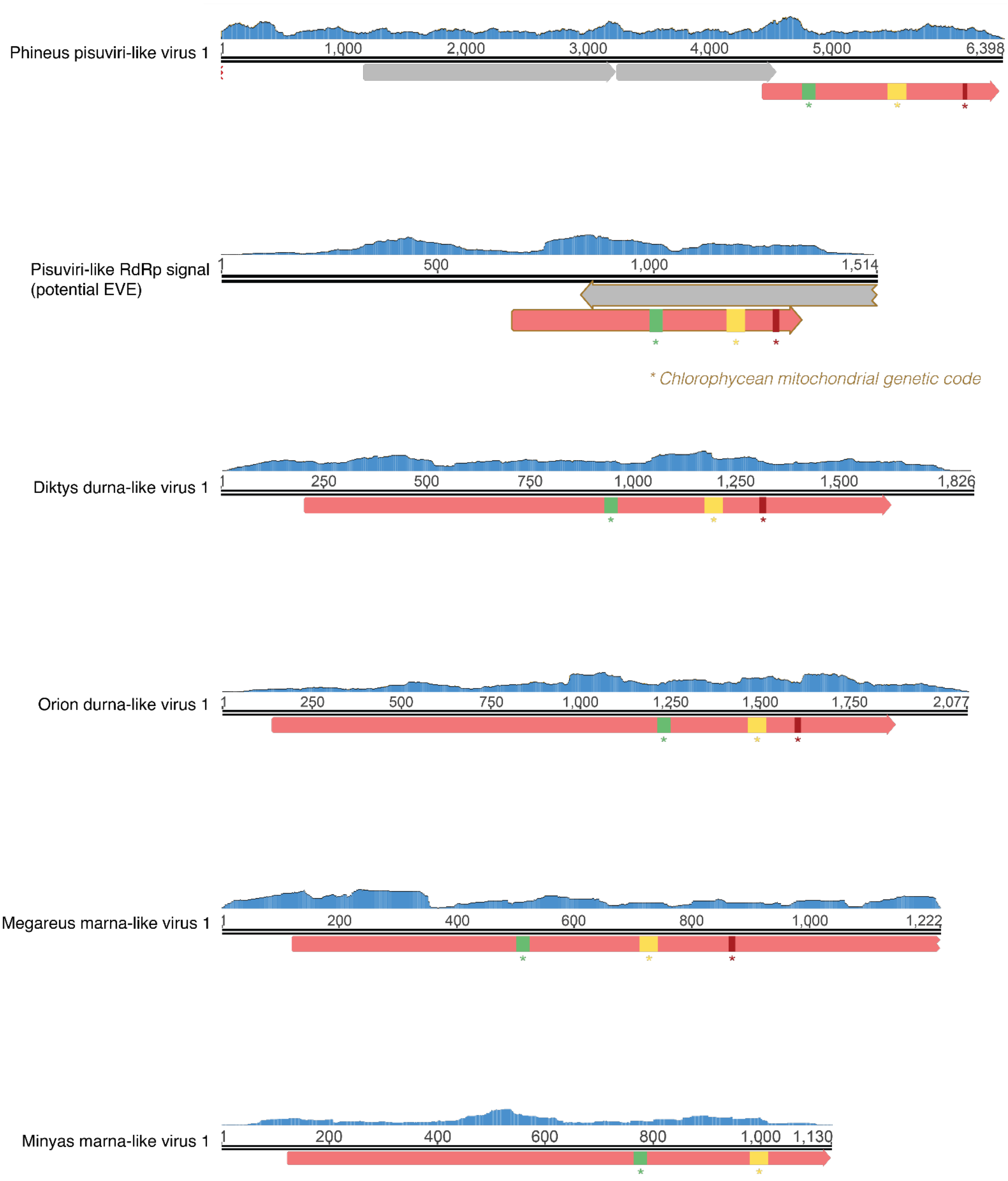

Figure A3. Genome organisation of the Pisuviricota-like viral signals identified in this study. Read coverage of each genome is represented as a blue histogram. ORFs were predicted using either standard genetic codes or the Chlorophycean mitochondrial code, and their directions represented as arrows. ORFs encoding RdRp-like signals and hypothetical functions are indicated in red and grey, respectively. A, B and C RdRp motifs are indicated in green, yellow and red boxes, respectively.
